# Wave energy and other environmental drivers as predictors of seeded-coral performance on the great barrier reef

**DOI:** 10.1038/s41598-025-22199-5

**Published:** 2025-11-03

**Authors:** Saskia Jurriaans, Carine D Lefèvre, Katie Allen, Christine Giuliano, Cathie A. Page, Marji Puotinen, Ben Radford, Carrie A. Sims, Taylor N. Whitman, Carly J. Randall

**Affiliations:** 1 Australian Institute of Marine Science, Townsville, QLD Australia; 2 Australian Institute of Marine Science, Perth, WA Australia; 3 Smithsonian Tropical Research Institute, Panama city, Panama; 4 College of Science and Engineering, James Cook University, Townsville, QLD Australia

**Keywords:** Current, Flow, Latitude, Recruitment, Restoration, Spawning, Spat, Marine biology, Restoration ecology

## Abstract

Wave energy shapes coral reef communities, yet its influence on early coral survival and growth remains poorly understood, limiting its use in reef restoration planning. This study investigated the survival and growth of three *Acropora* species deployed on seeding devices across a wave energy gradient at three reefs on the Great Barrier Reef. After 1.5-2 years, survival varied significantly within reefs, among sites, and among species, with highest average yield at Moore Reef (*A. millepora*, 32% after 554 days) followed by Davies Reef (*A. hyacinthus*, 24% after 527 days) and Heron Reef (*A. hyacinthus*: 13% and *A. cf. kenti*: 23% after 834 days). However, no single environmental variable, including nominal wave energy, bottom stress, flow velocity, sedimentation or benthic community composition consistently predicted survival, and effects weakened over time. Coral size and survival varied more at the device level than across sites, indicating the importance of fine-scale spatial and transient factors. These findings underscore the limitations of broad-scale environmental models to guide restoration and highlight the need for flexible, site-specific strategies. While seeding devices show promise as a scalable restoration tool, their success depends on matching species to suitable microhabitats and monitoring local conditions over time to support long-term outcomes.

**Supplementary Information:**

The online version contains supplementary material available at 10.1038/s41598-025-22199-5.

## Introduction

Wave energy plays a dominant role in shaping coral reef communities, influencing both their physical structure and biological composition^1^. Within-reef wave energy gradients drive spatial variation in benthic and fish communities, favouring fast-growing corals with compact, wave-resistant morphologies and encrusting crustose coralline algae in exposed areas^2,3^, while slower-growing corals with more complex morphologies and sediment-tolerant taxa, such as soft corals and fleshy macroalgae, dominate sheltered zones^4,5^. Wave energy also directly influences coral physiological performance, recruitment, and survival by influencing water motion that affects nutrient delivery, waste removal, larval dispersal, settlement cues, and exposure to physical disturbance^6–9^. As such, understanding wave energy gradients is critical for effective reef conservation and restoration planning.

With coral reefs increasingly threatened by climate change, particularly extreme heatwaves causing mass bleaching and mortality^10,11^, effective and scalable restoration strategies are urgently needed^12,13^. While reducing global carbon emissions remains the primary strategy to conserve coral reefs^14^, slow progress in achieving global emissions targets has necessitated additional adaptation actions^15^. As such, reef restoration, including the deployment of sexually-produced coral recruits, is increasingly recognised as a strategic approach, alongside emissions reduction and integrated ecosystem management, to enhance reef resilience and recovery^16,17^.

Traditional restoration methods, such as coral gardening, are limited by their reliance on donor colonies and challenges in up-scaling^18^. In contrast, sexually-produced corals offer greater potential for large-scale restoration, providing higher genetic diversity and the opportunity for selective breeding and assisted evolution to enhance climate resilience^19,20^. As a result, restoration programs that sexually produce corals are gaining momentum^21–23^. However, high post-settlement mortality is a primary bottleneck and challenge for coral restoration^21^ driven by species-specific responses to environmental variability^24–26^. To improve our understanding of natural recovery dynamics, and to achieve large-scale restoration success, we must better understand these specific drivers of post-settlement mortality.

The global distribution of *Acropora* species across tropical reefs, combined with their ecological importance as reef builders and framework species, makes them a globally relevant target for coral restoration^27^. Many *Acropora* taxa are now also listed on the International Union for the Conservation of Nature (IUCN) Red List of Threatened Species as vulnerable, near threatened, endangered or critically endangered. Understanding how environmental variability—particularly hydrodynamic conditions—shapes their early survival is critical to improving deployment strategies for these keystone species.

**Fig. 1:**
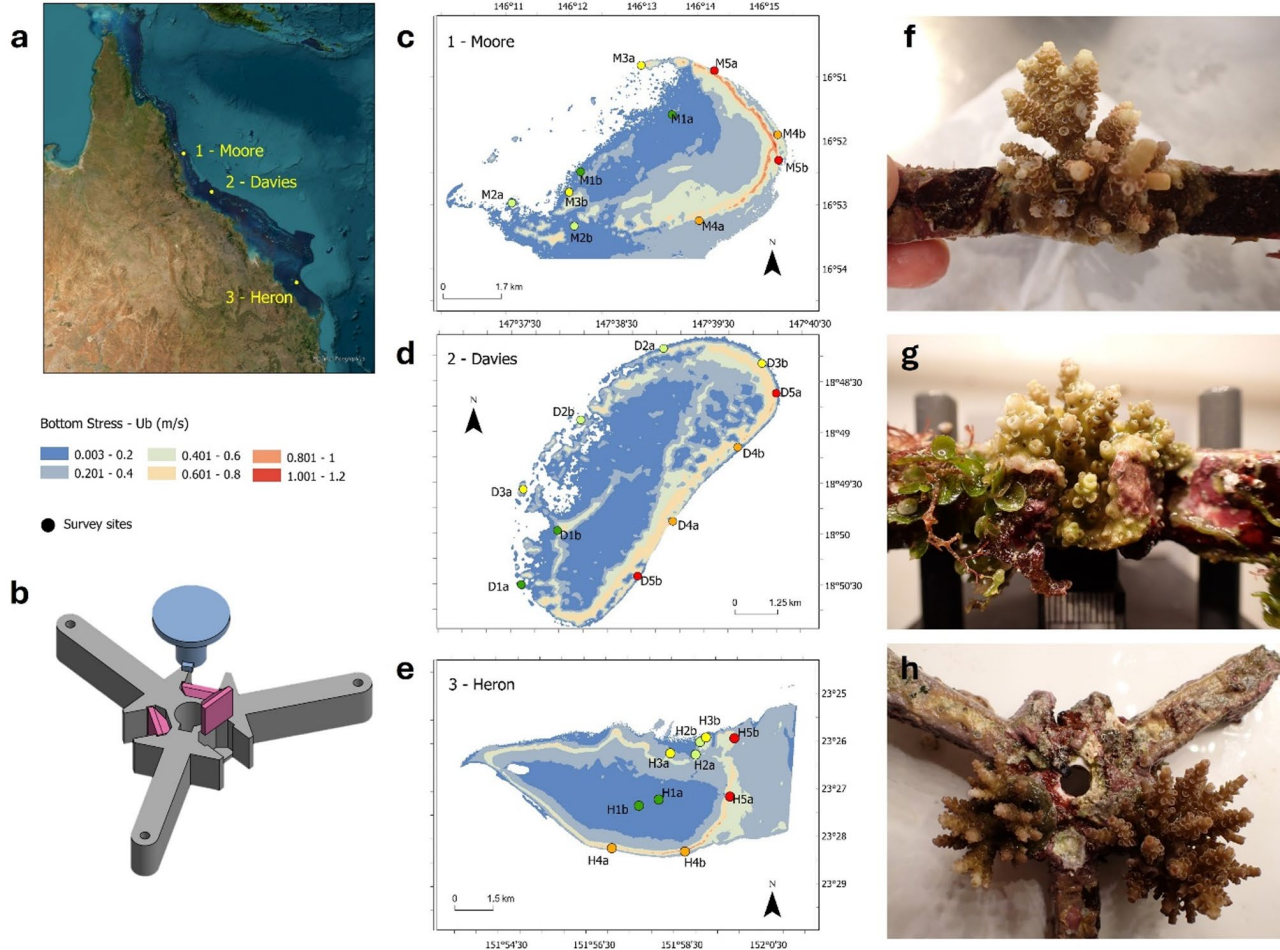
(**a**) Study area showing the locations of Moore, Davies, and Heron Reefs on the Great Barrier Reef. (**b**) Schematic of the coral seeding device. (**c**–**e**) Spatial distributions of long-term bottom stress used as a proxy for wave energy to guide site selection at Moore (**c**), Davies (**d**), and Heron (**e**). Sites were selected to span a wave energy gradient, with lower wave energy shown in blue and higher energy in red. At each reef, two sites were selected per wave energy level (levels 1 to 5), designated ‘a’ or ‘b’ (e.g., M1a, M1b). (**f**–**h**) Examples of surviving corals: *Acropora millepora* at Moore (**f**), *A. hyacinthus* at Davies (**g**), and *A. hyacinthus* and *A. cf. kenti* at Heron (**h**). Maps were generated using ArcGIS Pro software version 3.3, https://www.esri.com/en-us/arcgis/products/arcgis-pro/overview.

Here we assessed the survival and growth of three *Acropora* species deployed on engineered seeding devices across a wave energy gradient at three reefs spanning ~ 1,000 km along the Great Barrier Reef (GBR). Species differed by reef with *A. millepora* deployed on single-species devices at Moore Reef (northern GBR), *A. hyacinthus* deployed at Davies Reef (central GBR) and a combination of *A. hyacinthus* and *A. cf. kenti* deployed on multi-species devices at Heron Reef (southern GBR; Fig. 1). At each reef, 10 sites spanning five wave energy classes were selected, and 25 devices were deployed per site. Devices, each containing three seeded settlement tabs, were secured at 3–4.5 m depth and monitored approximately every three months for survival and growth over 18 months. To evaluate how environmental conditions across multiple spatial scales influenced post-settlement survival and growth, we combined high-resolution spatial habitat maps with in-situ monitoring of environmental variables, including flow velocity, temperature, sedimentation, and fine-scale benthic composition. Because reef habitats and environmental factors such as sedimentation and benthic community structure co-vary along wave energy gradients^1^, these variables were measured to explore whether they could independently explain variation in coral performance across sites, particularly as they have been shown to influence early post-settlement coral survival^28,29^. As different species were used at each reef, survival and growth data were analysed separately using Bayesian logistic and linear regressions. By identifying conditions that enhance or constrain coral survival on seeded devices, our findings provide practical guidance for tailoring species- and site-specific deployment strategies in coral restoration programs, with relevance for tropical reef restoration globally.

## Results

### Moore reef

**Fig. 2:**
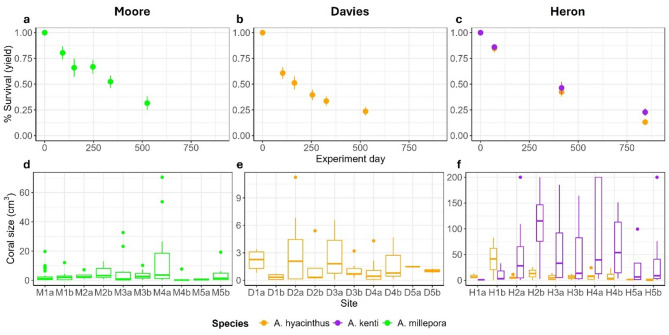
(**a**–**c**) Average yield (percentage of devices with at least one surviving coral) of *Acropora millepora*,* A. hyacinthus*, and *A. cf. kenti* over time at Moore, Davies, and Heron Reefs. Each point shows the mean across 10 sites per reef, with error bars representing standard error. (**d**–**f**) Distribution of coral size for each species across sites at Moore, Davies and Heron Reefs. Note the different scales on the y-axis.

After ~ 18 months (554 days) of deployment at Moore Reef, device-level survival (i.e., yield, percentage of devices with at least one surviving coral; 25 devices per site, 10 sites per reef) of *A. millepora* at Moore Reef averaged 32% ± 7% s.e.m. (range: 8–76%, *n* = 10 sites; Fig. 2a), while tab-level survival (i.e., percentage of individual settlement tabs with at least one survival coral; 3 tabs per device, 25 devices per site) was 14% ± 4% s.e.m. (*n* = 10 sites). Survival varied significantly over time and across environmental variables (Supplementary Materials, Table S1), declining at all sites but remaining highest at low wave-energy sites (M1a, M1b; 62% after 554 days) and lowest at moderately exposed sites (M3; 8% after 554 days).

**Fig. 3:**
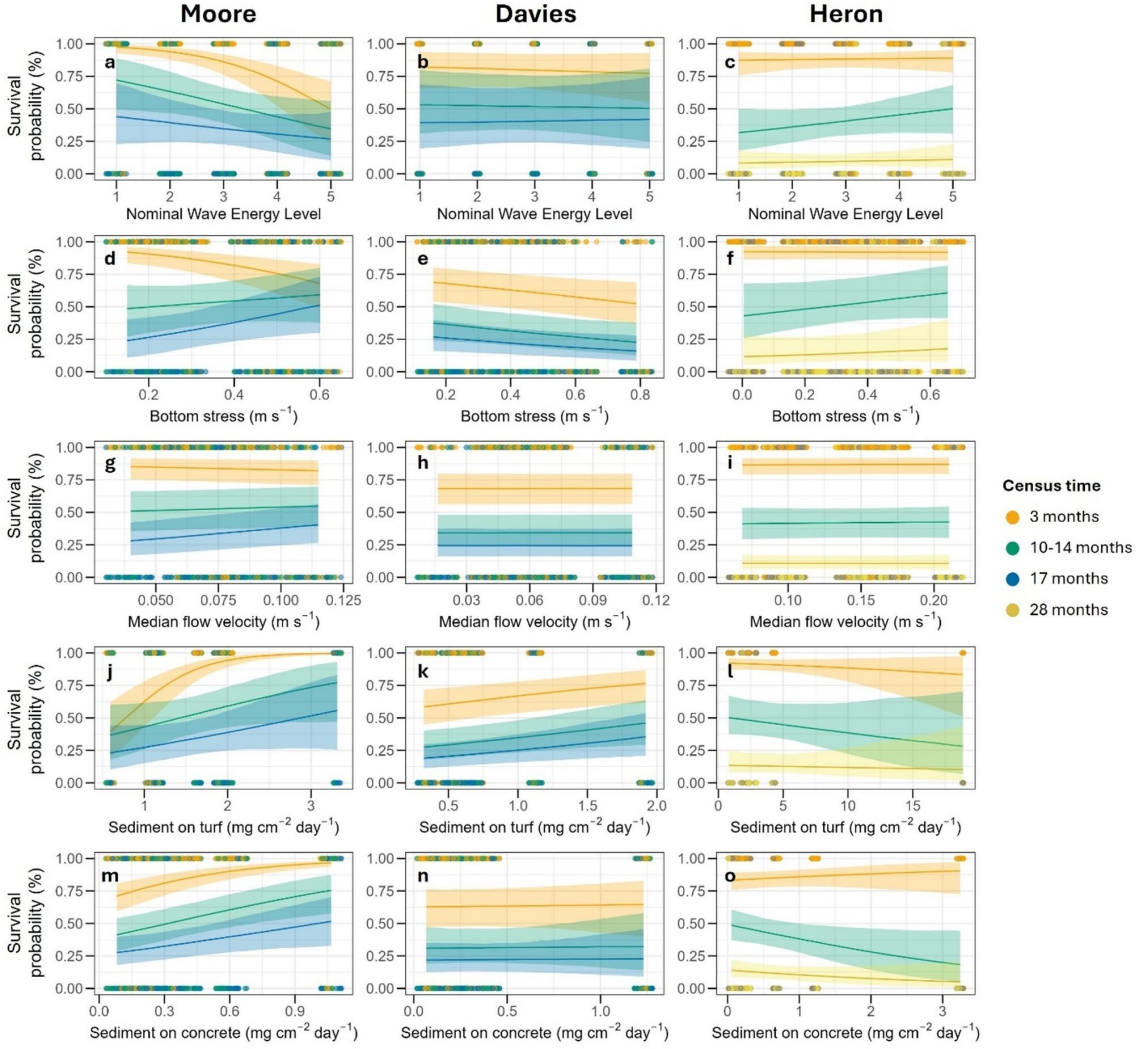
Predicted survival probability (%) across Moore, Davies, and Heron Reefs in relation to: (**a**–**c**) nominal wave energy level predicted by spatial models; (**d**–**f**) long-term bottom stress predictions; (**g**–**i**) median flow velocity measured in-situ at each site; (**j**–**l**) sedimentation on turf pods; (**m**–**o**) sedimentation on concrete sed pods. Lines represent the predicted survival probabilities, and the shaded areas indicate 95% credible intervals. Data points are coloured by census time.

Survival decreased with increasing nominal wave energy and bottom stress during the first three months, with weaker responses to flow velocity (Fig. 3). Median flow velocities, measured at ~ 50 cm above the substrate using current meters, ranged from 0.04 to 0.12 m s^−1^ but did not consistently align with nominal wave-energy categories (Fig. S1). Bottom stress predictions (0.15–0.60 m s^−1^) generally exceeded measured flow velocities (Fig. S1). Survival increased with higher sediment deposition, measured using SedPods (coral surface mimics) and TurfPods (algal turf mimics), on both turf and concrete substrates (Fig. 3), although this effect weakened over time and was absent at the final census (Table S1). TurfPods captured more sediment (1.06–2.01 mg cm^−2^ day^−1^) than concrete SedPods (0.16–0.64 mg cm^−2^ day^−1^), with the highest deposition at sheltered sites (M1a, M1b; Fig. S1).

**Fig. 4:**
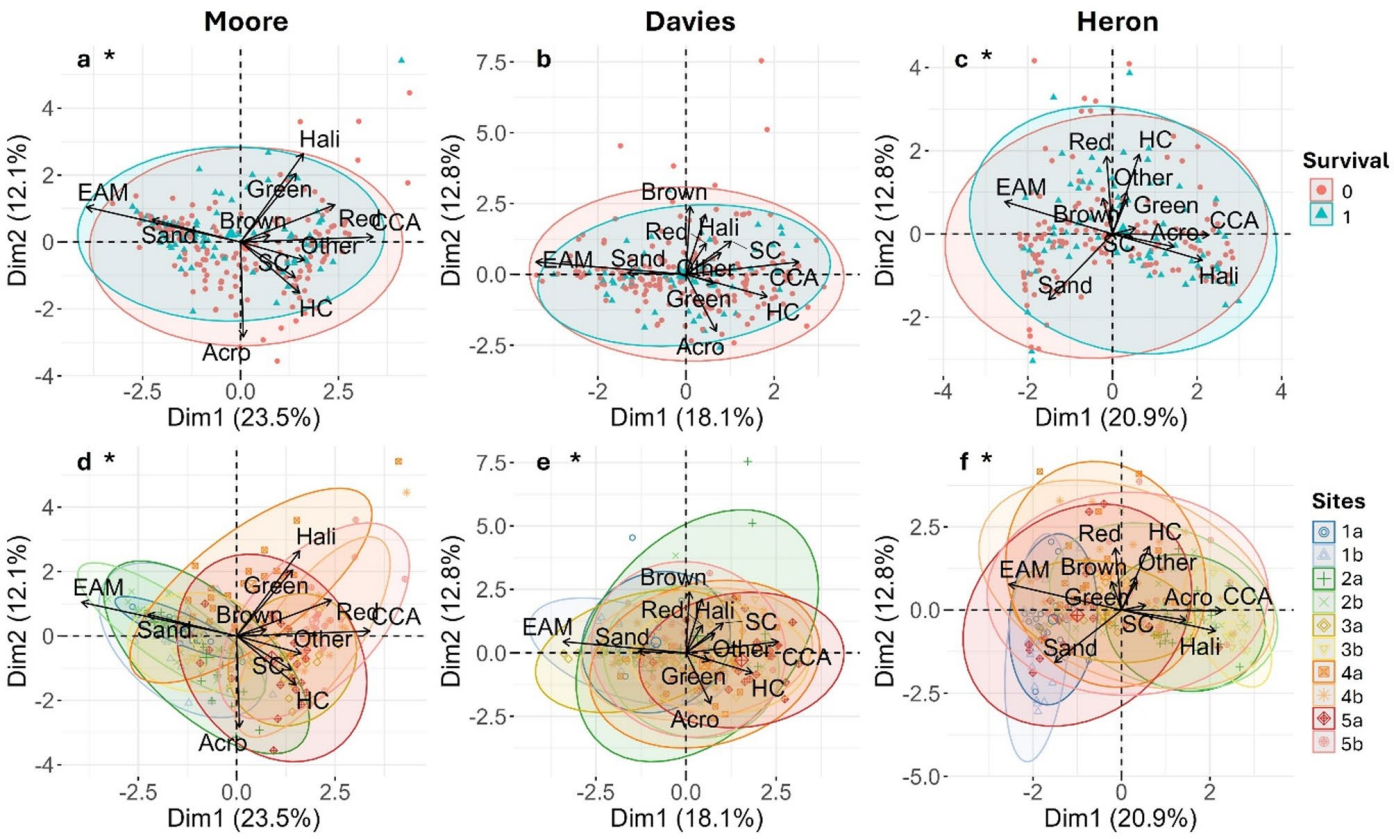
Principal component analysis (PCA) of the benthic community composition across Moore, Davies and Heron Reefs. (a-c) PCA biplots showing variation in benthic community composition by yield; (d-f) PCA biplots showing variation across sites. Data points represent individual devices with ellipses representing the 95% confidence intervals. Plots marked with an asterisk (*) indicate significant effects of survival or site on benthic community composition (PERMANOVA, *p* < 0.05). *Abbreviations*: CCA = crustose coralline algae and cyanobacteria, EAM = epilithic algal matrix, Acro = *Acropora* colonies, Brown = brown macroalgae, Green = green macroalgae, Red = red macroalgae, Hali = *Halimeda*, HC = non-*Acropora* hard coral, SC = soft coral, Other = primarily sponges, ascidians, zoanthids, and hydroids.

Benthic community composition, assessed from quadrat images analysed using ReefCloud^30^, differed significantly when grouped by survival (Fig. 4a), although the effect size was small (PERMANOVA, R^2^ = 0.011, *p* = 0.014). Survival decreased with increasing *Acropora* and soft coral cover but increased with epilithic algal matrix (EAM; Fig. S2, Table S2). Community composition also varied significantly across sites (R^2^ = 0.288, *p* = 0.001), driven by differences in EAM, crustose coralline algae (CCA), cyanobacteria, and *Acropora* cover (Fig. 4d).

Coral size averaged 4.65 cm^3^ (s.d. = 9.55, *n* = 121) after 554 days, with the largest colonies at M4a (13.16 cm^3^, s.d. = 19.90, *n* = 18) and the smallest at M5a (0.59 cm^3^, s.d. = 0.49, *n* = 6; Fig. 2b). Size varied more within than between sites, and no environmental predictors significantly explained coral size at the final census (Fig. S3; Table S3).

### Davies reef

After ~ 17 months (527 days) of deployment at Davies Reef, *A. hyacinthus* yield averaged 24% ± 4% s.e.m. (range: 4–52%, *n* = 10 sites), with tab-level survival averaging 9% ± 1% s.e.m. (*n* = 10 sites; Fig. 2b). Survival declined sharply in the first 103 days, appearing most pronounced at high wave-energy sites (D5a, D5b). However, models using nominal wave energy did not provide strong evidence for an effect of wave energy on survival (Fig. 3b; Table S1). Similarly, survival showed no clear effect of flow velocity (Fig. 3h), although a stronger negative effect was detected for bottom stress (Fig. 3e). Median flow velocity ranged from 0.02 to 0.11 m s^−1^, with no clear relationship to nominal wave energy (Fig. S1). Predicted bottom stress (0.16–0.79 m s^−1^) was ~ 8 times higher than in-situ flow velocity. Higher sedimentation on TurfPods increased survival, but sedimentation on concrete SedPods had no effect (Fig. 3). TurfPods collected more sediment (0.32–1.92 mg cm^−2^ day^−1^) than SedPods (0.07–1.23 mg cm^−2^ day^−1^), with the highest accumulation at low-energy sites (Fig. S1).

Benthic community composition did not predict survival (PERMANOVA, *p* = 0.947; Fig. 4b), but varied across sites (R2 = 12.7%, *p* = 0.001; Fig. 4e), driven primarily by EAM, CCA, cyanobacteria, and brown macroalgae.

Coral size averaged 1.75 cm^3^ (s.d. = 2.11, *n* = 68) after 527 days (Fig. 2b), with the largest colony at D2a (11.26 cm^3^) and smallest at D1b (0.004 cm^3^). Size varied more within than between sites, with no significant effects of environmental predictors (Fig. S3; Table S3).

### Heron reef

Survival at Heron Reef varied significantly over time (27 months) and across sites (*n* = 10) but did not differ between *A. cf. kenti* and *A. hyacinthus* (Fig. 2; Table S4). Initial survival was high for both species (85–86% after three months), declining to an average yield (± s.e.m.) of 23 ± 4% for *A. cf. kenti* and 13 ± 1% for *A. hyacinthus* after 27 months. Bayesian logistic regressions showed that survival varied more within than between sites, as indicated by greater variance at the device level (1.07, 95% CI: 0.84–1.32) compared to the site level (0.63, 95% CI: 0.31–1.11). Wave energy, bottom stress, and flow velocity did not significantly predict survival (Table S4), although species-specific interaction with bottom stress was detected. Survival of *A. cf. kenti* increased with bottom stress (Estimate = 1.14, 95% CI: 0.25–2.02), corresponding to a threefold increase in odds of survival, while *A. hyacinthus* showed no clear response (Fig. 3). Median flow velocities were highest and most variable at exposed sites, and closely correlated with bottom stress (range 0.01–0.66 m s^− 1^, Fig. S1; Pearson’s *r* = 0.79, df = 18, *p* < 0.01). Sediment accumulation on concrete SedPods negatively affected *A. hyacinthus* survival at later timepoints (Estimate = -0.63, 95% CI: -1.09 to -0.18), while sedimentation on TurfPods had no effect (Fig. 3). Sediment loads were highest at lagoonal and moderate wave-energy sites, with turf accumulating substantially more than concrete (Fig. S1).

Benthic community composition was weakly associated with survival (R^2^ = 0.009, *p* = 0.032; Fig. 4c), with higher survival linked to *Halimeda* and non-*Acropora* hard coral cover (Table S2, Fig. S4). Community composition varied significantly across sites (PERMANOVA, *p* = 0.001; Fig. 4f), driven by EAM, CCA, cyanobacteria, and *Halimeda* on PC1, and hard coral, red macroalgae, and sand on PC2.

After 834 days, *A. cf. kenti* grew ~ 14 times larger than *A. hyacinthus* (average size ± s.d. respectively, 112.02 ± 358.09 cm^3^ (*n* = 60) vs. 8.09 ± 14.64 cm^3^ (*n* = 36); Bayesian linear regression, Estimate = 1.44, 95% CI: 0.76–2.10; Fig. 2b). Growth varied more within than between sites, with little variation explained by environmental predictors (Table S5). For *A. hyacinthus*, sedimentation on concrete negatively affected growth (log-odds estimate: -1.03, 95% CI: -2.05 to -0.01; Fig. S3), suggesting that increasing sedimentation is associated with a 64% reduction in the odds of growth (odds ratio of 0.36).

## Discussion

This study provides new insights into the environmental and spatial drivers influencing early survival and growth of corals seeded on engineered devices across three reefs on the GBR. While survival varied considerably among sites, species, and through time, no single environmental factor consistently predicted long-term outcomes. Importantly, survival and growth were shaped more by fine-scale conditions at the device level than by broader environmental gradients, aligning with another recent study demonstrating that early post-settlement survival was influenced most heavily at spatial scales < 1 m^29^.

Early post-settlement survival is a major bottleneck to coral recovery, but seeding reefs with sexually-produced settlers on engineered devices shows promise, with device-level survival (yield) in this study ranging from 13% to 32% after 17 to 27 months, comparable to similar trials on the GBR and elsewhere using *Acropora* species^29,31,32^. Direct comparisons to wild recruit survival are limited due to the difficulty of detecting newly settled spat in natural conditions, particularly during the critical early post-settlement period when mortality is highest^33^. However, the lack of clear relationships between broadscale hydrodynamic metrics (nominal wave energy, bottom stress, and median flow velocity) and long-term survival highlights the limitations of using spatial models to guide site selection for coral seeding. Instead, localised environmental heterogeneity and transient events, such as episodic sedimentation or short-lived disturbances, likely play a more important role.

Hydrodynamic factors negatively influenced early survival (first 3 months) but were not a long-term predictor of mortality (> 1 year). This possibly reflects the vulnerability of newly settled spat to shear stress, dislodgement, and sediment scouring^34,35^. These effects diminished as corals grew larger, highlighting the value of timing deployments to coincide with calmer periods to improve initial survival, noting that practical constraints such as limited settlement space and the annual timing of coral spawning may limit this approach. To improve restoration outcomes, long-term, fine-scale hydrodynamic monitoring should be integrated into deployment planning, alongside adaptive management strategies, such as flexible site selection and iterative (re)deployment of seeded devices. However, we acknowledge that the feasibility of such monitoring may be limited at scale due to cost and logistical constraints and may be best suited to targeted deployments or as part of pilot-phase assessments.

The relationship between sedimentation and survival was highly site- and species-specific. In some cases, higher sediment deposition at low-energy sites initially correlated with better survival, perhaps due to reduced algal competition^36^. However, these benefits were temporary, with prolonged deployment at sediment-heavy sites resulting in declines in survival and growth, particularly for *A. hyacinthus* at Heron Reef. Sediment can have deleterious effects on newly settled corals by smothering spat, impeding light availability and interfering with key physiological processes^37–39^. It can also impose significant energetic costs. For example, in adult corals, mucus production for sediment rejection has been shown to increase from 35% to 65% of daily respiration under sediment exposure, potentially diverting energy away from growth and calcification^40^. Despite these known effects, reported outcomes vary. Some studies found no significant impact of sedimentation on survival^41^, and others observed negative effects on growth and survival limited to the first month post-settlement^42^. We speculate that sediment characteristics and species life-history traits may help explain some of these inconsistencies. For instance, fine, nutrient-rich sediments may have stronger detrimental effects by increasing turbidity and inducing hypoxia with prolonged exposure^43^, while species with higher lipid reserves may be better equipped to tolerate short-term sedimentation stress. Our results highlight the importance of considering both short-term benefits and long-term risks of sedimentation when selecting deployment sites. Further research into sediment grain size, nutrient content, and resuspension rates could improve predictions of sedimentation impacts and provide valuable insights for site selection in reef restoration efforts.

Benthic community composition at the device level explained little variation in survival, reinforcing that even within-site conditions were too variable to capture clear community-survival relationships at this scale. However, at Moore Reef, *Acropora* abundance negatively affected survival, likely due to competition, shading, or allelopathic effects^44^ with nearby colonies occurring within ~ 50 cm of the devices. In contrast, EAM presence was positively associated with survival. This finding is somewhat unexpected, as EAM is often considered a competitor and barrier to coral settlement and early survival^36,45,46^. However, this positive association may reflect nuances in how EAM was classified in our study, as our scoring captured a mixture of short and longer turfs, and areas where turf co-occurred with CCA, rather than exclusively long, sediment-laden turfs typically associated with negative effects^47^. Additionally, because the devices sat on top of the EAM, spat were growing above rather than within the turf, likely reducing direct competition. At Heron Reef, *Halimeda* algae and non-*Acropora* corals positively influenced survival, possibly by reducing grazing pressure^48^ as accidental grazing by herbivorous fishes can cause significant declines in spat survival^32^. These results highlight the value of site-specific knowledge of benthic assemblages when identifying suitable seeding locations, but also reinforces that benthic cover alone is not sufficient for predicting survival. Other fine-scale factors such as micro-refugia^49^ and CCA overgrowth^50^ have been shown to play a more direct role in determining survival outcomes.

An important consideration for large-scale reef restoration is whether to deploy single-species or multispecies devices to maximise efficiency and resilience. Deploying multiple species on a single device may hedge against the risk of poor survival if local conditions are suboptimal for a particular species, which is particularly valuable in spatially and temporally variable environments where species respond differently to factors such as flow, sedimentation, and light availability^1^. However, multispecies devices could also introduce competitive interactions as corals grow. We tested multispecies devices at Heron Reef, where *Acropora cf. kenti*, a habitat generalist, consistently outperformed *A. hyacinthus*, a species more specialised for high-light, high-energy habitats^51^. After 27 months, only 3% of devices supported both species, limiting our ability to directly assess interspecific competition. While *A. cf. kenti* had a higher mean yield (23 ± 4%) than *A. hyacinthus* (13 ± 1%), species was not a significant predictor of yield in our model. Nonetheless, the observed difference may reflect the broader ecological niche of *A. cf. kenti* compared to *A. hyacinthus*^27^ and highlights the potential value of incorporating species’ ecological niches into site selection decisions. Future trials should explicitly test single- versus multi-species designs, particularly as corals grow larger and competitive dynamics emerge. This includes evaluating whether higher yields are best achieved through multi-species devices or through multi-species deployments using single-species devices, to determine whether species diversity enhances resilience or if species-specific targeting is more effective.

Although this study focused on three *Acropora* species on the GBR, the findings have broader relevance for restoration of tropical reef systems globally. *Acropora* species dominate coral cover across Indo-Pacific reefs^27^ and often play keystone roles in reef structure and ecosystem function^52^. The environmental and spatial factors influencing early survival and growth in this study, particularly the dominance of fine-scale processes, are likely relevant to other coral species and reef environments. To translate this into practical guidance, we recommend that site selection be based on in-situ assessments of local environmental conditions rather than solely on spatial models or maps. These site assessments should incorporate indicators of microhabitat suitability, including fine-scale sedimentation rates, benthic community composition, and hydrodynamics conditions. Such assessments could be conducted using short-term deployments of sensor arrays, rapid benthic surveys or pilot seeding trials. Species selection can then be tailored to these habitat characteristics. While environmental drivers may differ across reefs, the overarching principle remains: tailoring site selection and deployment strategies to local conditions, and allowing flexibility to respond to unexpected events, will be critical for improving the success and scalability of coral seeding as a restoration tool.

While this study provides valuable insights into coral survival on seeded devices for reef restoration, several limitations should be noted. Hydrodynamic and sedimentation measurements were constrained by instrument failures and logistical challenges, restricting our ability to capture seasonal variability and extreme events. In-situ flow velocities also did not align well with modelled nominal wave energy, reflecting the difficulty of using long-term probability data to predict short-term, fine-scale conditions in patchy reef habitats. Additionally, important environmental variables, such as light, nutrients, and microhabitat complexity were not measured but likely influenced survival. Early survival patterns were not predictive of longer-term outcomes, highlighting the need for extended monitoring and more standardized temporal sampling across sites. Finally, the low number of devices supporting multiple species limited our ability to assess interspecific interactions.

Together, our findings underscore the importance of fine-scale spatial and temporal processes in shaping early coral survival and growth on seeded devices. Broadscale environmental models provided limited guidance for site selection, with local conditions and species-specific responses dominating outcomes. As large-scale coral seeding programs expand globally, integrating fine-scale environmental monitoring, flexible site selection, and adaptive management will be essential for improving restoration success. Testing species- and habitat-specific deployment strategies across reef types and regions, while ensuring long-term monitoring to capture delayed responses, will further refine best practices and support more effective ecosystem restoration.

## Materials and methods

### Coral collection to device assembly

Approximately twelve gravid colonies of *Acropora millepora*, *A. hyacinthus*, and *A. cf. kenti* were collected in mid-November 2021 from Moore, Davies, and Heron Reefs (Fig. 1a) and transported by boat to the National Sea Simulator (SeaSim) at the Australian Institute of Marine Science (AIMS) in Townsville (Queensland, Australia). Corals were held in outdoor holding tanks under reef-specific temperatures and natural light. Spawning occurred several days after the full moon (November 19, 2021) with gametes collected, cross-fertilised within reefs, and reared to larvae following Pollock et al.^53^. Four to nine days post-fertilisation, competent larvae settled on pre-conditioned concrete settlement sheets, each containing 400 individual settlement tabs designed by AIMS to facilitate mass settlement and deployment in engineered seeding devices. Settled spat were maintained under flow-through filtered seawater at natal reef temperatures, low light^50^ and fed daily with enriched *Artemia*, rotifers, and microalgae. Symbiont uptake was facilitated by co-culturing with donor colonies.

Seeding devices (Fig. 1b) made of 95% Alumina ceramic (Shanghai Gongtao Ceramics Co., Ltd. PRC) and designed by AIMS, allowed the deployment of three seeded tabs per device. Devices were designed with protrusions to provide partial protection from grazing and predation of spat by fish^32^. Devices were seeded with *A. millepora* for Moore Reef (2 tabs with 2–4 spat, 1 tab with > 4 spat), *A. hyacinthus* for Davies Reef (only tabs with > 4 spat), and a combination of *A. cf. kenti* and *A. hyacinthus* for Heron Reef (only tabs with > 4 spat). Spat densities were standardised as much as possible, prioritising tabs with > 4 spat (> 1 per 40 mm^2^), although densities varied by species and reef. Assembled devices were held on steel rods in indoor aquaria for up to 15 days prior to deployment (see Table S6 for details on spawning, settlement, and deployment).

### Experimental design

Devices were deployed across three reefs spanning ~ 7° latitude along the GBR. At each reef, 10 sites spanning a wave-energy gradient were selected using high-resolution spatial maps (LIDAR with Sentinel 2 satellite data), benthic habitat prediction models^54^, and wave-energy modelling^55^. Sites were selected to ensure similar depth (6 ± 2 m), hardbottom substrate, and the presence of live coral. Wave energy was approximated using a bottom stress metric (Ub) derived from numerical models at 10m resolution^55^, which captured spatial variability in routine wave conditions across each reef. Sites expected to be too rough to access were excluded. A generalised random tessellation stratified design^56^ ensured unbiased and spatially balanced site selection, with reserve sites available for substitution if in-situ conditions were unsuitable. The final sites were labelled with a systematic code: the first letter of the reef (M for Moore, D for Davies, H for Heron), followed by numbers 1 to 5 for wave-energy class (low to high), and ‘a’ or ‘b’ for within-class replicates (Fig. 1c-e). See Supplement S1 for details on the site selection method.

At each site, 25 devices were deployed at ~ 4 m depth (range: 3–4.5 m depth at the lowest astronomical tide) along a 25 m transect, secured to the reef using zip-ties. One-month-old spat were deployed at Heron Reef, while 2-month-old spat were deployed at Moore Reef and Davies Reef (Table 1). Vessel transport times ranged from 4 to 6 days, with minimal transport mortality (< 3% across reefs; Table S7). Devices were censused approximately every three months, although timing varied across sites and reefs due to logistical constraints (Table 1). Spat survival was assessed by counting live, discrete coral colonies on each tab. Upon retrieval, the size of surviving corals was measured along three axes: maximum length, perpendicular length, and maximum height. Colony size (cm^3^) was estimated as the product of these dimensions.

**Table 1:** Deployment, census and retrieval metadata for *Acropora millepora*,* A. hyacinthus*, and *A. hyacinthus* and *A. cf. kenti*, highlighting differences in census timing and deployment duration across reefs.

Reef	Species	Deployment date	Retrieval date	Days deployed	Census days
Moore	*Acropora millepora*	Feb 2022	Aug 2023	554	86–92, 148–152*, 243–248*, 333–338
Davies	*Acropora hyacinthus*	Feb 2022	Jul 2023	527	100–103, 163–166, 253–256, 322–326
Heron	*Acropora hyacinthus; A. cf. kenti*	Jan 2022	May 2024	834	71–75, 415–420

* Not all sites were censused.

### Environmental predictors

Three metrics were used to describe site hydrodynamics: (1) nominal wave energy categories (1–5) used to guide site selection, (2) long-term bottom stress predictions (Ub) for each site’s waypoint, and (3) in-situ flow velocity recorded at 1-minute intervals using drag-tilt current meters (Marotte HS, James Cook University), mounted ~ 50 cm above the substrate. Current meters were retrieved, cleaned, and redeployed during each census trip.

Additional site-level environmental variables were measured, including sedimentation and benthic community composition. Temperature was also recorded, at 10-minute intervals using HOBO Water Temp Pro v2, to assess potential variation among sites, but was excluded from analyses due to minimal variability (< 0.2 °C; Table S8).

Sediment accumulation was measured using SedPods (coral surface mimics) and TurfPods (algal turf mimics), deployed horizontally in triplicate at each site for 4 to 11 days. After collection, sediment was filtered, dried (60 °C, 48 h), and weighed, with accumulation calculated in mg cm^−2^ day^−1^. Full pod design and deployment details are in Supplement S1.

Benthic community composition was assessed at deployment using 1 m^2^ quadrat images taken around each device (Olympus Tough TG-6), analysed with ReefCloud^30^. Of 50 points per image, 15 were classified manually and 35 by an AI model^57^. Benthic categories included: *Acropora*, non-*Acropora* hard coral, soft coral, sand, epilithic algal matrix (EAM), crustose coralline algae (CCA), cyanobacteria, macroalgae (green, brown, red, *Halimeda*), and ‘other’ (sponges, ascidians, zoanthids, hydroids). Model performance (F-scores) ranged from 0.57 (sand) to 0.82 (*Acropora* and EAM; Table S9).

### Data analyses

Device-level survival (presence/absence of a surviving coral) was modelled separately for each reef using Bayesian logistic regression^58^. To evaluate the role of individual environmental variables, we fit separate models in which each predictor (wave energy, bottom stress, median flow velocity, sedimentation, benthic composition) was included as a fixed factor alongside time, and with site as a random factor. For Heron Reef, species was also included as a fixed factor, and device as a random factor. We considered an effect “significant” if the 95% credible interval of the posterior distribution did not include zero. See Supplement S1 for full model specifications, MCMC settings, and diagnostics.

The relationship between coral size (log-transformed) and environmental variables was assessed using Bayesian linear regression models, with site and device as random factors. Environmental predictors were included as fixed factors, with species included for Heron Reef. Model diagnostics followed the same procedures as above.

Flow velocity data were not available for the entire period at all sites due to lost meters, so analyses were restricted to periods with overlapping data. Median flow velocity was chosen as predictor in the model, as it correlated with the 10th and 90th percentile, and standard deviation (Table S10).

Benthic community composition was reduced via Principal Component Analysis (PCA) to identify key benthic groups driving variation across sites. Differences in benthic composition between sites and survival groups were tested using PERMANOVA, and survival probability was modelled as a function of key benthic groups using generalised linear models with a binomial distribution and logit link function. See Supplement S1 for detailed methods regarding PCA.

Statistical analyses and figure creation were performed in *R* v3.6.2^59^, packages are detailed in Supplement S1.

## Supplementary Information

Below is the link to the electronic supplementary material.

Supplementary Material 1

## Data Availability

Data is available through AIMS data repository ( [https://doi.org/10.25845/9PAE-ZZ03](https:/doi.org/10.25845/9PAE-ZZ03) ); scripts are available via Github (SaskiaJurriaans/CAD-Year1).
