# Supplementary material for: Wave energy and other environmental drivers as predictors of seeded-coral performance on the great barrier reef
Source: Sci Rep. 2025 Nov 3;15:38335. doi: 10.1038/s41598-025-22199-5 (PMC12583707; doi:10.1038/s41598-025-22199-5)
Supplement: Supplementary file 1 — Supplementary Material 1 [file 41598_2025_22199_MOESM1_ESM.pdf]

Supplementary Materials

Wave energy and other environmental drivers as predictors of seeded-coral performance on the Great Barrier Reef

Saskia Jurriaans, Carine D Lefèvre, Katie Allen, Christine Giuliano, Cathie A Page, Marji Puotinen, Ben Radford, Carrie Sims, Taylor N Whitman, Carly J Randall

Table S1

Table S1. Summary of Bayesian logistic regression models examining the effects of time and environmental drivers on **coral survival** at **Moore Reef** and **Davies Reef.** Each model includes a random intercept for site, fixed effects for time and the environmental predictors, and their interaction. Environmental predictors include nominal wave energy level (wave), bottom stress (Ub), flow velocity (flow), sedimentation on turfpods (turf) and sedimentation on sedpods (concrete). The posterior mean estimates indicate the expected change in log-odds of survival and the credible intervales reflect the uncertainty in the posterior distributions. Asterisk indicates significant effects as CI does not include zero. For Moore Reef, census t2 and t3 were not included in the model because not all sites were surveyed due to bad weather.

Model	Effects type	Parameter	Moore Reef		Davies Reef	
			Estimate	95% CI	Estimate	95% CI
**Survival** ~ time + (1|Site)	Fixed	Intercept (t1)	1.61	(0.93, 2.26)*	0.56	(-0.03, 1.16)
		t2	-	-	-0.48	(-0.84, -0.11)*
		t3	-	-	-1.02	(-1.40, -0.63)*
		t4	-1.47	(-1.91, -1.05)*	-1.32	(-1.70, -0.93)*
		t5	-2.24	(-2.71, -1.79)*	-1.79	(-2.18, -1.40)*
	Random	Site	0.88	(0.51, 1.54)*	0.87	(0.52, 1.48)*
**Survival** ~ time * wave + (1|Site)	Fixed	Intercept (t1)	4.62	(3.12, 6.23)*	1.66	(0.52, 2.98)*
		t2	-	-	-0.46	(-1.04, 0.09)
		t3	-	-	-1.25	(-1.84, -0.66)*
		t4	-3.25	(-4.31, -2.22)*	-1.46	(-2.06, -0.86)*
		t5	-4.65	(-5.70, -3.56)*	-2.09	(-2.69, -1.50)*
		wave	-0.93	(-1.43, -0.50)*	-0.08	(-0.38, 0.24)
	Interaction	wave * t2	-	-	-0.01	(-0.20, 0.18)
		wave * t3	-	-	0.08	(-0.12, 0.27)
		wave * t4	0.52	(0.23, 0.81)*	0.05	(-0.15, 0.24)
		wave * t5	0.73	(0.44, 1.02)*	0.11	(-0.10, 031)
	Random	Site	0.81	(0.42, 1.50)*	1.28	(0.61, 2.44)*
**Survival** ~ time * Ub + (1|Site)	Fixed	Intercept (t1)	2.29	(2.01, 3.88)*	0.97	(0.27, 1.63)*
		t2	-	-	-0.47	(-0.90, -0.04)*
		t3	-	-	-1.04	(-1.46, -0.61)*
		t4	-3.23	(-4.08, -2.39)*	-1.32	(-1.75, -0.89)*
		t5	-4.59	(-5.44, -3.77)*	-1.81	(-2.28, -1.35)*
		Ub	-3.73	(-5.57, -1.83)*	-1.10	(-2.03, -0.18)*
	Interaction	Ub * t2	-	-	-0.04	(-0.60, 0.50)
		Ub * t3	-	-	0.05	(-0.51, 0.60)
		Ub * t4	4.77	(2.81, 6.76)*	-0.00	(-0.58, 0.58)
		Ub * t5	6.49	(4.49, 8.61)*	0.06	(-0.49, 0.63)
	Random	Site	0.98	(0.55, 1.71)*	0.79	(0.45, 1.34)*

**Survival** ~ time * flow + (1|Site)	Fixed	Intercept (t1)	1.87	(1.23, 2.55)*	0.78	(0.24, 1.37)*
		t2	-	-	-0.58	(-0.91, -0.23)*
		t3	-	-	-1.13	(-1.50, -0.79)*
		t4	-1.92	(-2.44, -1.42)*	-1.43	(-1.78, -1.07)*
		t5	-3.12	(-3.66, -2.60)*	-1.90	(-2.28, -1.53)*
		flow	-3.10	(-5.12, -1.07)*	0.00	(-0.95, 0.98)
	Interaction	flow * t2	-	-	0.01	(-0.96, 0.97)
		flow * t3	-	-	0.04	(-0.95, 1.05)
		flow * t4	5.18	(1.46, 8.93)*	0.04	(-0.93, 0.99)
		flow * t5	10.66	(6.98, 14.44)*	0.01	(-0.96, 0.98)
	Random	Site	0.94	(0.55, 1.61)*	0.87	(0.51, 1.47)*
**Survival** ~ time * turf + (1|Site)	Fixed	Intercept (t1)	-1.79	(-3.08, -0.56)*	0.18	(-0.43, 0.80)
		t2	-	-	-0.49	(-0.91, -0.10)*
		t3	-	-	-1.02	(-1.44, -0.59)*
		t4	0.85	(-0.04, 1.72)	-1.31	(-1.73, -0.86)*
		t5	0.28	(-0.66, 1.21)	-1.80	(-2.24, -1.36)*
		turf	2.30	(1.62, 3.00)*	0.52	(0.15, 0.90)*
	Interaction	turf * t2	-	-	0.01	(-0.18, 0.20)
		turf * t3	-	-	0.00	(-0.18, 0.19)
		turf * t4	-1.65	(-2.21, -1.09)*	-0.02	(-0.21, 0.17)
		turf * t5	-1.77	(-2.33, -1.21)*	0.01	(-0.18, 0.20)
	Random	Site	0.83	(0.46, 1.46)*	0.78	(0.47, 1.31)*
**Survival** ~ time * concrete + (1|Site)	Fixed	Intercept (t1)	0.69	(0.16, 1.24)*	0.52	(-0.14, 1.21)
		t2	-	-	-0.47	(-0.89, -0.04)*
		t3	-	-	-1.02	(-1.45, -0.59)*
		t4	-1.17	(-1.54, -0.79)*	-1.33	(-1.76, -0.90)*
		t5	-1.75	(-2.15, -1.37)*	-1.79	(-2.27, -1.35)*
		concrete	2.53	(1.96, 3.09)*	0.06	(-0.78, 0.88)
	Interaction	concrete*t2	-	-	-0.02	(-0.51, 0.46)
		concrete*t3	-	-	0.01	(-0.49, 0.51)
		concrete*t4	-1.02	(-1.71, -0.28)*	0.00	(-0.50, 0.51)
		concrete*t5	-1.48	(-2.21, -0.76)*	-0.02	(-0.53, 0.51)
	Random	Site	0.69	(0.36, 1.25)*	0.86	(0.52, 1.44)*

Table S2

Table S2. Summary of logistic regressions on the impact of various key benthic groups on coral survival. The coefficients (Estimates) represent the effect size of each variable on survival, with standard errors, z values, and p-values provided to indicate statistical significance. Significant predictors ($p < 0.05$) are marked with *.

Definitions: CCA/Cyano = CCA and cyanobacteria grouped together, EAM = epilithic algal matrix, Acro = Acropora colonies, BrownMA = brown macroalgae, HaliMA = Halimeda, Hard coral = non-Acroporid hard corals.

Reef	Variable	Estimate	Std. Error	z value	p-value	Significance
Moore	CCA/Cyano	-0.35	0.31	-1.12	0.26	n.s.
	EAM	1.21	0.46	2.61	0.01	*
	Acro	-4.74	1.81	-2.62	0.01	*
	BrownMA	-15.44	18.58	-0.83	0.41	n.s.
	HaliMA	2.65	2.46	1.08	0.28	n.s.
	Sand	0.11	1.94	0.06	0.96	n.s.
	Hard Coral	0.28	1.30	0.21	0.83	n.s.
	Soft Coral	-3.49	1.33	-2.62	0.01	*
Davies	CCA/Cyano	-0.26	0.41	-0.62	0.54	n.s.
	EAM	0.13	0.62	0.21	0.83	n.s.
	Acro	0.63	1.11	0.57	0.57	n.s.
	BrownMA	2.46	16.37	0.15	0.88	n.s.
	HaliMA	-8.73	9.71	-0.90	0.37	n.s.
	Sand	-0.15	3.12	-0.05	0.96	n.s.
	Hard Coral	-0.45	1.24	-0.37	0.72	n.s.
	Soft Coral	0.48	3.49	0.14	0.89	n.s.
Heron	CCA/Cyano	0.41	0.38	1.07	0.29	n.s.
	EAM	-0.48	0.53	-0.91	0.37	n.s.
	Acro	-0.97	1.19	-0.81	0.42	n.s.
	BrownMA	-25.58	32.00	-0.80	0.42	n.s.
	HaliMA	2.71	1.06	2.56	0.01	*
	Sand	-1.84	0.94	-1.95	0.05	n.s.
	Hard Coral	4.25	1.81	2.35	0.02	*
	Soft Coral	0.75	3.00	0.25	0.80	n.s.

Table S3

Table S3. Summary of Bayesian mixed-effects models examining the relationship between **coral size** (log-transformed) and environmental predictors at **Moore Reef** and **Davies Reef**. Models include random effects for site and device. The environmental predictors are nominal wave energy level (wave), bottom stress (Ub), flow velocity (flow), sedimentation on turfpods (turf), sedimentation on sedpods (concrete) and the principal component axes (PC1 and PC2) of the redundancy plot, which relates community composition and environmental drivers. Estimates (Est.), standard errors (Std. Error), and 95% credible intervals (CI) are reported for each predictor. Significant effects (i.e., credible intervals that do not include zero) are marked with an asterisk (*).

Model	Effects type	Moore Reef			Davies Reef		
		Est.	Std. Error	95% CI	Est.	Std. Error	95% CI
**Size_log** ~ (1|Site) + (1|Device)	Intercept	1.10	0.16	(0.77, 1.41)*	0.79	0.11	(0.57, 0.99)*
	Site SD	0.35	0.19	(0.03, 0.77)*	0.18	0.13	(0.01, 0.5)*
	Device SD	0.54	0.15	(0.16, 0.78)*	0.26	0.13	(0.02, 0.5)*
**Size_log** ~ wave + (1|Site) +	Wave	-0.03	0.12	(-0.29, 0.19)	-0.03	0.1	(-0.22, 0.17)
	Intercept	1.21	0.41	(0.45, 2.02)*	0.87	0.3	(0.27, 1.47)*
	Site SD	0.40	0.21	(0.06, 0.9)*	0.2	0.14	(0.01, 0.53)*
	Device SD	0.54	0.14	(0.2, 0.78)*	0.26	0.13	(0.02, 0.51)*
**Size_log** ~ Ub + (1|Site) + (1|Device)	Ub	-0.71	1.09	(-3.05, 1.35)	-0.3	0.73	(-1.7, 1.19)
	Intercept	1.34	0.41	(0.54, 2.18)*	0.9	0.29	(0.3, 1.45)*
	Site SD	0.39	0.20	(0.08, 0.85)*	0.19	0.14	(0.01, 0.53)*
	Device SD	0.54	0.14	(0.19, 0.77)*	0.26	0.13	(0.02, 0.51)*
**Size_log** ~ flow + (1|Site) + (1|Device)	Flow	-1.04	4.13	(-9.21, 7.35)	0.23	3.03	(-5.75, 6.09)
	Intercept	1.19	0.35	(0.47, 1.88)*	0.78	0.22	(0.33, 1.21)*
	Site SD	0.36	0.19	(0.04, 0.82)*	0.19	0.13	(0.01, 0.49)*
	Device SD	0.54	0.14	(0.2, 0.78)*	0.26	0.14	(0.01, 0.51)*
**Size_log** ~ turf + (1|Site) + (1|Device)	Turf	0.01	0.23	(-0.45, 0.49)	0.22	0.26	(-0.29, 0.75)
	Intercept	1.09	0.43	(0.2, 1.9)*	0.62	0.23	(0.14, 1.09)*
	Site SD	0.41	0.21	(0.07, 0.88)*	0.19	0.13	(0.01, 0.51)*
	Device SD	0.54	0.14	(0.19, 0.78)*	0.26	0.07	(0.41, 0.7)*
**Size_log** ~ concrete + (1|Site) + (1|Device)	Concrete	0.24	0.61	(-0.88, 1.52)	0.33	0.36	(-0.38, 1.07)
	Intercept	1.00	0.31	(0.38, 1.59)*	0.69	0.16	(0.38, 0.99)*
	Site SD	0.41	0.20	(0.08, 0.89)*	0.19	0.14	(0.01, 0.53)*
	Device SD	0.54	0.14	(0.21, 0.78)*	0.26	0.13	(0.02, 0.51)*
**Size_log** ~ PC1 + PC2 + (1|Site) + (1|Device)	PC1	0.94	0.87	(-0.79, 2.68)	-0.45	0.69	(-1.8, 0.9)
	PC2	-0.67	0.84	(-2.33, 0.92)	-0.35	0.64	(-1.54, 0.89)
	Intercept	1.04	0.17	(0.69, 1.39)*	0.79	0.12	(0.54, 1.02)*
	Site SD	0.42	0.18	(0.13, 0.83)*	0.20	0.14	(0.02, 0.53)*
	Device SD	0.45	0.15	(0.08, 0.71)*	0.27	0.08	(0.42, 0.71)*

Table S4

Table S4. Summary of Bayesian logistic regression models examining the effects of time and environmental drivers on **coral survival** at **Heron Reef.** Each model includes a random intercept for site and device, fixed effects for time, species and the environmental predictors, and their interactions. Environmental predictors include nominal wave energy level (wave), bottom stress (Ub), flow velocity (flow), sedimentation on turfpods (turf) and sedimentation on sedpods (concrete). The posterior mean estimates indicate the expected change in log-odds of survival and the credible intervales reflect the uncertainty in the posterior distributions. Asterisk indicates significant effects as CI does not include zero.

Model	Effects type	Parameter	Heron Reef	
			**Estimate**	**95% CI**
**Survival** ~ Spp * time + (1|Site) + (1|Device)	Fixed	Intercept (t1)	2.17	(1.61, 2.75) *
		t2	-2.56	(-2.97, -2.14) *
		t3	-4.5	(-4.99, -4) *
		Species	0.1	(-0.34, 0.54)
	Interaction	Species * time	0.11	(-0.47, 0.69)
	Random	Site	0.63	(0.31, 1.11) *
		Device	1.07	(0.84, 1.32) *
**Survival** ~ Spp * time * wave + (1|Site) + (1|Device)	Fixed	Intercept (t1)	1.9	(0.84, 2.92) *
		t2	-2.87	(-3.67, -2.1) *
		t3	-4.39	(-5.3, -3.51) *
		Species	-0.03	(-0.63, 0.57)
		wave	0.04	(-0.27, 0.36)
	Interaction	wave * Species	0.1	(-0.12, 0.32)
		wave * t2	0.15	(-0.09, 0.39)
		wave * t3	0.04	(-0.23, 0.31)
		Species * t2	0.02	(-0.69, 0.78)
		Species * t3	0.19	(-0.61, 0.91)
		wave * Spp * t2	-0.04	(-0.32, 0.24)
		wave * Spp * t3	0.06	(-0.22, 0.37)
	Random	Site	0.55	(0.24, 1.04) *
		Device	1.05	(0.81, 1.29) *
**Survival** ~ Spp * time * Ub + (1|Site) + (1|Device)	Fixed	Intercept (t1)	2.53	(1.84, 3.44) *
		t2	-2.78	(-3.41, -2.16) *
		t3	-4.55	(-5.24, -3.88) *
		Species	-0.06	(-0.64, 0.52)
		Ub	-0.1	(-1.03, 0.82)
	Interaction	Ub * Species	1.14	(0.25, 2.02) *
		Ub * t2	1.19	(-0.48, 2.75)
		Ub * t3	0.85	(-1.09, 2.77)
		Species * t2	-0.25	(-1.18, 0.69)

		Species * t3	0.15	(-1, 1.24)
		Ub * Spp * t2	0.13	(-2.01, 2.29)
		Ub * Spp * t3	0.52	(-2.21, 3.25)
	Random	Site	0.82	(0.37, 1.61) *
		Device	1.05	(0.81, 1.30) *
**Survival** ~ Spp * time * flow + (1|Site) + (1|Device)	Fixed	Intercept (t1)	1.84	(1.27, 2.44) *
		t2	-2.23	(-2.74, -1.73) *
		t3	-3.94	(-4.52, -3.35) *
		Species	0.32	(-0.22, 0.83)
		flow	0.24	(-1.73, 2.23)
	Interaction	flow * Species	0.45	(-1.41, 2.34)
		flow * t2	0.13	(-1.84, 2.08)
		flow * t3	-0.39	(-2.24, 1.47)
		Species * t2	-0.31	(-1.01, 0.36)
		Species * t3	0.18	(-0.54, 0.89)
		flow * Spp * t2	0.48	(-1.93, 3.67)
		flow * Spp * t3	0.21	(-2.14, 3.11)
	Random	Site	0.58	(0.29, 1.04) *
		Device	1.00	(0.77, 1.24) *
**Survival** ~ Spp * time * turf + (1|Site) + (1|Device)	Fixed	Intercept (t1)	2.52	(1.92, 3.25) *
		t2	-2.45	(-2.84, -2.06) *
		t3	-4.34	(-4.78, -3.9) *
		Species	0.16	(-0.27, 0.58)
		turf	-0.05	(-0.15, 0.06)
	Interaction	turf * Species	0	(-0.08, 0.09)
		turf * t2	-0.01	(-0.09, 0.07)
		turf * t3	0.03	(-0.07, 0.12)
		Species * t2	0.11	(-0.52, 0.75)
		Species * t3	0.63	(-0.05, 1.3)
		turf * Spp * t2	-0.03	(-0.14, 0.09)
		turf * Spp * t3	-0.05	(-0.18, 0.08)
	Random	Site	0.65	(0.29, 1.28) *
		Device	1.05	(0.82, 1.29) *
**Survival** ~ Spp * time * concrete + (1|Site) + (1|Device)	Fixed	Intercept (t1)	1.61	(1.11, 2.15) *
		t2	-1.64	(-2.11, -1.17) *
		t3	-3.4	(-3.96, -2.84) *
		Species	0.94	(0.36, 1.52) *
		concrete	0.2	(-0.24, 0.65)
	Interaction	concrete * Species	-0.63	(-1.09, -0.18) *
		concrete * t2	-0.66	(-1.16, -0.18) *
		concrete * t3	-0.57	(-1.21, 0.04)
		Species * t2	-0.58	(-1.33, 0.15)
		Species * t3	-0.26	(-1.06, 0.51)
		concrete * Spp * t2	0.04	(-0.71, 0.78)
		concrete * Spp * t3	0.16	(-0.79, 1.06)
	Random	Site	0.45	(0.13, 0.90) *

		Device	1.00	(0.76, 1.24) *

Table S5

Table S5. Summary of Bayesian mixed-effects models examining the relationship between **coral size** (log-transformed) and environmental predictors at **Heron Reef.** Models include random effects for site and device and fixed effect for species (Spp). The environmental predictors are nominal wave energy level (wave), bottom stress (Ub), flow velocity (flow), sedimentation on turfpods (turf), sedimentation on sedpods (concrete) and the principal component axes (PC1 and PC2) of the redundancy plot, which relates community composition and environmental drivers. Estimates, standard errors (Std. Error), and 95% credible intervals (CI) are reported for each predictor. Significant effects (i.e., credible intervals that do not include zero) are marked with an asterisk (*).

Model	Effects	Heron Reef		
		**Estimate**	**Std. Error**	**95% CI**
**Size_log** ~ Spp + (1|Site) + (1|Device)	Species	1.44	0.34	(0.76, 2.1)*
	Intercept	1.62	0.30	(1.03, 2.22)*
	Site SD	0.31	0.22	(0.01, 0.83)*
	Device SD	0.94	0.39	(0.07, 1.54)*
**Size_log** ~ wave * Spp + (1|Site) + (1|Device)	Wave	-0.24	0.22	(-0.66, 0.19)
	Species	0.39	0.87	(-1.31, 2.15)
	Wave * Species	0.31	0.24	(-0.16, 0.79)
	Intercept	2.44	0.77	(0.87, 3.88)*
	Site SD	0.34	0.25	(0.02, 0.94)*
	Device SD	0.92	0.42	(0.08, 1.56)*
**Size_log** ~ Ub * Spp + (1|Site) + (1|Device)	Ub	-1.08	1.47	(-4.03, 1.88)
	Species	0.76	0.70	(-0.62, 2.12)
	Ub * Species	1.83	1.70	(-1.62, 5.24)
	Intercept	2.03	0.61	(0.83, 3.25)*
	Site SD	0.35	0.26	(0.01, 1)*
	Device SD	0.93	0.40	(0.08, 1.54)*
**Size_log** ~ flow * Spp + (1|Site) + (1|Device)	Flow	-2.14	3.73	(-9.42, 5.45)
	Species	1.15	0.67	(-0.12, 2.44)
	Flow * Species	1.87	4.02	(-6.1, 10.11)
	Intercept	1.94	0.60	(0.76, 3.13)*
	Site SD	0.33	0.25	(0.02, 0.92)*
	Device SD	0.96	0.40	(0.1, 1.58)*
**Size_log** ~ turf * Spp + (1|Site) + (1|Device)	Turf	-0.04	0.05	(-0.15, 0.06)
	Species	1.52	0.42	(0.72, 2.38)*
	Turf * Species	-0.03	0.06	(-0.16, 0.09)
	Intercept	1.77	0.38	(1.02, 2.54)*
	Site SD	0.29	0.23	(0.01, 0.86)*
	Device SD	0.94	0.40	(0.08, 1.55)*
**Size_log** ~ concrete * Spp + (1|Site) + (1|Device)	Concrete	0.22	0.33	(-0.41, 0.86)
	Species	1.78	0.39	(1, 2.53)*
	Concrete * Species	-1.03	0.53	(-2.05, -0.01)*
	Intercept	1.54	0.35	(0.85, 2.23)*

	Site SD	0.32	0.25	(0.01, 0.94)*
	Device SD	0.91	0.40	(0.07, 1.54)*
**Size_log** ~ (PC1 * Spp) + (PC2 * Spp) + (1|Site) + (1|Device)	PC1	-0.29	1.96	(-4.13, 3.62)
	PC2	-0.59	1.81	(-4.22, 2.82)
	Species	1.41	0.36	(0.72, 2.11)*
	PC1 * Species	1.23	2.36	(-3.29, 5.81)
	PC2 * Species	-1.13	2.09	(-5.22, 3.04)
	Intercept	1.62	0.31	(1.02, 2.21)*
	Site SD	0.32	0.24	(0.01, 0.92)*
	Device SD	0.96	0.41	(0.1, 1.6)*

Supplement S1: Supporting Information for Material and Methods

Deployment site selection

High-resolution (10 m) spatial maps of Moore, Davies, and Heron Reef were created to identify sites comparable in coral habitat (i.e. hardbottom), depth, and that spanned a wave-energy gradient. These maps were generated by merging LIDAR with Sentinel 2 satellite data to produce detailed bathymetry for each reef. A spatial benthic habitat prediction model [1] was then employed to determine the probability of coral habitat existence within a specific depth range (6 ± 2 m tidal range). Afterward, routine significant wave height (Hs) data at the 90th percentile, modelled at a 10 m resolution with a numerical wave model [2] were integrated, and sites that were expected to be too rough to visit were excluded from the selection process.

Wave-energy gradients under routine conditions were assessed using a bottom stress metric (Ub) modelled at a 10-m resolution with a numerical wave model [2]. This metric facilitated the identification of wave-energy variations across the three reefs, which were categorised into five classes. To ensure equal representation and spatial balance across the study areas, a generalised random tessellation stratified (GRTS) sampling design [3] was employed. Using this approach, 30 sites (10 + 20 reserves) were identified at each reef, spanning five bottom-stress classes. The advantage of using the GRTS for site selection is that the method is unbiased and reproducible. Furthermore, this approach allowed for the inclusion of statistically valid reserve sites within the design. These reserve sites provided flexibility for rejecting certain locations and incorporating new ones if *in situ* assessments deviated from the model predictions, such as the absence of hardbottom or variations in depth outside the target range. Furthermore, an additional requirement of each deployment site was the presence of live coral; if this was absent, reserve sites were used. A total of 10 sites were ultimately selected per reef, and these were labelled using a systematic code: the first letter of the reef name (M for Moore, D for Davies, H for Heron), followed by numbers 1 to 5 indicating the nominal wave-energy levels form low to high, and 'a' or 'b' to distinguish between duplicate sites within each wave-energy level.

Quantification of sediment accumulated on SedPods and TurfPods

SedPods: PVC ring (9 cm diameter, 7 cm high) filled with concrete to mimic coral surfaces [4]

TurfPods: Same as SedPods but with 5 mm artificial grass to mimic algal turfs [5].

Deployment:

- Davies Reef: 12-23 July 2022 (11 days), 7-14 April 2023 (7 days)
- Heron Reef: 27 February – 3 March 2023 (4 days)
- Moore Reef: 16-20 April 2023 (4 days)

Laboratory processing:

Following field collection, pods were rinsed with MilliQ water with sediments collected in individual jars and left to settle for 24 hours before decanting the top water. The remaining sediment was filtered onto pre-combusted (450°C, 4h) 47 mm Whatman GF/C filters (1.2 µm pore size) using a vacuum manifold. Filters were rinsed three times with MilliQ water, wrapped in pre-combusted aluminium foil and dried at 60°C for 48 hours. Filters were then weighed to 0.00001 g to determine total sediment loads gravimetrically. To correct for residual salts, 'wet filter blanks' (WFB) were used, with the average WFB weight applied at each location. Result are sediment concentration (mg cm^{-2} day^{-1}) for particulate matter > 1.2 µm.

Calculation: Total net accumulated sediments = [(final filter weight - initial filter weight) – WFB] / Surface area / Days of deployment

Where initial filter weight = unused filter weight (mg), final filter weight = loaded filter weight (mg), WFB = averaged wet filter blank weight (mg), surface area = 67.93 cm^2 (SedPod/TurfPod).

Data analyses

Coral survival

Device-level survival (presence/absence of at least one surviving coral) was modeled separately for each reef using Bayesian logistic regression. Models were constructed for each environmental predictor, including nominal wave energy (site classification), bottom stress (Ub), median flow velocity, sedimentation on turf and concrete, and benthic community composition. Environmental variables and time were included as fixed factors, with site as a random factor. For Heron Reef, species was also included as a fixed factor, and device was included as a random factor to account for species-level variation.

Bayesian models were implemented using the 'brms' package [6] via RStan [7], assuming a Bernoulli distribution with a logit link function. Each model ran three MCMC chains with 5000 iterations, thinned to a rate of 5, with a warm-up of 1000 iterations. Convergence was assessed using trace plots, autocorrelation plots, Rhat values, and effective sample sizes. Models were validated using DHARMa residual diagnostics [8] and visualized using 'ggplot2' [9]. Effects were considered significant if credible intervals excluded zero.

Coral size

Coral size was modelled using Bayesian linear regression models with a normal distribution using the 'brms' package. Size was log-transformed to meet model assumptions of normality and homoscedasticity. Separate models were developed for each reef, with site and device as random factors and the environmental predictors as fixed factors. For Heron Reef, species was included as a fixed factor. Model convergence, MCMC mixing, and posterior predictive checks were assessed using the same diagnostic methods described above.

Environmental predictors

Flow velocity data were not available for the entire period at all sites due to lost meters, so analyses were restricted to periods with overlapping data. At Heron Reef, data from January-March 2022 were used, while at Davies Reef, data from February-April and July-October 2022 were used. For Moore Reef, data were available for all sites except two (M1a and M1b) from February-April 2022, and for all sites except two (M5a and M5b) from February-April 2023. Because hydrodynamic patterns for sites with data from both 2022 and 2023 were similar, it allowed for combined analysis by averaging across years. Rose plots, frequency plots, boxplots, and histograms were used to assess the distribution of flow velocities.

Pearson's correlation tests showed that 10th and 90th percentile flows were significantly positively correlated with median flow (Table S4); thus, median flow was chosen as the predictor used in survival models.

Similarly, temperature data were analysed for overlapping periods at each reef. At Heron and Davies reefs, a window of 14 months was used, while at Moore Reef, data from January to July 2023 were analysed. Minimum, maximum, and average temperatures varied by less than 0.2°C across sites (Supplementary materials Table S8), so temperature was excluded from the final models.

Benthic community composition was analysed using point count data from ReefCloud converted to percent cover. Principal Component Analysis (PCA) reduced data dimensionality and identified key benthic groups explaining variance in the community composition amongst sites and between plots with and without survivors. PCA results were visualised using the 'factoextra' package [10]. Permutational Multivariate Analysis of Variance (PERMANOVA) was performed using the 'adonis2' function from the 'vegan' package [11] to assess differences in benthic community composition between sites or survival groups. Generalised linear models (GLMs) with a binomial distribution and logit link function were then used with the 'glm' function from base R to model survival probability as a function of key benthic groups identified by the PCAs. Relationships were visualised using 'ggplot2' scatter plots with logistic regression lines. Lastly, Redundancy Analyses (RDA) were performed using the 'vegan' package to understand the main gradients of variation in community composition influenced by environmental variables. Ordination diagrams were created to visualise the relationships between the benthic communities and environmental variables using the 'envfit' function of the 'vegan' package. The 'envfit' function fits environmental vectors onto the ordination space to assess the strength and significance of relationships between environmental variables and the community composition. The significance of these relationships was tested using permutation tests with 999 iterations. Results were visualised as vectors in the ordination plots, where the direction and length of each vector indicate the strength and alignment of each variable with the principal components.

Table S6

Table S6. Summary of coral broodstock collection, spawning, larval settlement, and deployment dates across Heron, Davies, and Moore Reefs. DAFM = Days After Full Moon. *Ahya = Acropora hyacinthus, Aken = Acropora cf. kenti, Amil = Acropora millepora.*

**Reef**	**Collection date**	**Species (No. colonies)**	**Spawning date**	**DA FM**	**Colonies for mass cultures**	**Mass cultures**	**Date settlement**	**Date deployment**
Moore	13-14 Nov	*Amil* (12)	28 Nov	9	7	1 x 70L	2 Dec	6-8 Feb ’22
	2021	*Aken* (10)	23 Nov	4	7	1 x 500L	2 Dec	Not deployd
Davies	17-20 Nov	*Ahya* (15)	28 Nov	9	7	2 x 500L	2 Dec	9-10 Feb ’22
	2021	Aken (12)	24 Nov	5	10	2 x 500L	2 Dec	Not deployd
Heron	9 Nov	*Ahya* (13)	2 Dec	13	4	2 x 70L	6 Dec	6-7 Jan ’22
	2021	*Aken* (12)	28 Nov	9	5	1 x 70L	6 Dec	6-7 Jan ’22

Table S7

Table S7. Transportation mortality after 3 days and 6 days on board vessel. Only devices deployed at Davies Reef were in transit for longer than 3 days.

Reef	Recruits alive pre-deployment	Recruits alive after 3 days	Recruits alive after 6 days	Sum mortality
Moore	97	96		1
Davies	188	188	188	0
Heron	228	214		6

Table S8

Table S8. Monthly temperature data for Moore Reef, Davies Reef, and Heron Reef from January 2022 to July 2023. For each reef, the table presents the number of sites where temperature was recorded (N), the mean temperature across sites, the standard deviation across sites, and the minimum and maximum temperatures recorded each month.

	Moore Reef					Davies Reef					Heron Reef				
**Date**	**N**	**Mean**	**SD**	**Min**	**Max**	**N**	**Mean**	**SD**	**Min**	**Max**	**N**	**Mean**	**SD**	**Min**	**Max**
2022-01											10	27.4	0.2	27.2	27.9
2022-02						10	29.3	0.1	29.2	29.5	10	27.3	0.1	27.1	27.5
2022-03						10	29.3	0.0	29.2	29.3	10	27.2	0.1	27.1	27.4
2022-04						10	28.3	0.0	28.2	28.3	10	26.0	0.2	25.8	26.2
2022-05	3	27.0	0.1	26.9	27.1	10	26.9	0.0	26.8	27.0	10	24.6	0.1	24.4	24.7
2022-06	3	25.7	0.2	25.5	25.9	10	25.3	0.1	25.1	25.4	10	22.1	0.3	21.4	22.4
2022-07	5	24.4	0.2	24.1	24.6	10	23.4	0.1	23.2	23.5	10	20.7	0.4	20.0	21.2
2022-08	4	24.8	0.1	24.7	25.0	9	23.5	0.0	23.5	23.5	10	21.0	0.2	20.8	21.2
2022-09	4	26.1	0.1	25.9	26.1	9	25.1	0.0	25.0	25.1	10	21.9	0.2	21.7	22.2
2022-10	8	27.8	0.2	27.6	28.0	9	27.0	0.0	27.0	27.1	10	23.3	0.3	23.0	23.9
2022-11	8	28.5	0.1	28.4	28.6	10	28.1	0.0	28.0	28.1	10	24.4	0.2	24.2	24.8
2022-12	8	29.0	0.0	29.0	29.1	10	28.7	0.0	28.7	28.8	10	25.5	0.2	25.3	25.8
2023-01	10	29.1	0.0	29.1	29.2	10	28.9	0.1	28.7	28.9	10	26.3	0.1	26.2	26.6
2023-02	10	29.1	0.0	29.1	29.2	9	28.9	0.0	28.9	28.9					
2023-03	10	29.0	0.0	29.0	29.1	9	28.7	0.0	28.7	28.8					
2023-04	10	29.0	0.0	28.9	29.1	9	28.6	0.0	28.5	28.6					
2023-05	10	27.0	0.2	26.7	27.3	9	26.3	0.1	26.1	26.4					
2023-06	10	26.1	0.1	26.0	26.3	9	25.4	0.0	25.3	25.4					
2023-07	10	25.7	0.2	25.5	26.0	9	25.2	0.1	25.1	25.3					

Table S9

Tabe S9. F1 scores to assess model performance based for benthic classification using ReefCloud. The F1-score considers precision and recall and indicates the model's ability to correctly classify each benthic category. Higher scores denote better classification performance.

Category	F1 Score
Acropora	0.82
EAM	0.82
Hard Coral	0.73
Sof Coral	0.70
CCA	0.64
Sand	0.57

Table S10

Table S10. Pearson correlation coefficients (r) and 95% confidence intervals between site-level hydrodynamic metrics across reefs.

**Reef**	**Variable pair**	**r**	**95% CI**	**p-value**
Moore	Median speed vs 90th percentile	0.898	0.742 – 0.962	<0.001
	Median speed vs 10th percentile	0.552	0.115 – 0.810	0.018
	Median speed vs stdev	0.810	0.551 – 0.926	<0.001
Heron	Median speed vs 90th percentile	0.971	0.877-0.993	<0.001
	Median speed vs 10th percentile	0.954	0.812-0.989	<0.001
	Median speed vs stdev	0.910	0.656-0.979	<0.001
Davies	Median speed vs 90th percentile	0.954	0.813 – 0.989	<0.001
	Median speed vs 10th percentile	0.957	0.824 – 0.990	<0.001
	Median speed vs stdev	0.958	0.827 – 0.990	<0.001

Figure S1

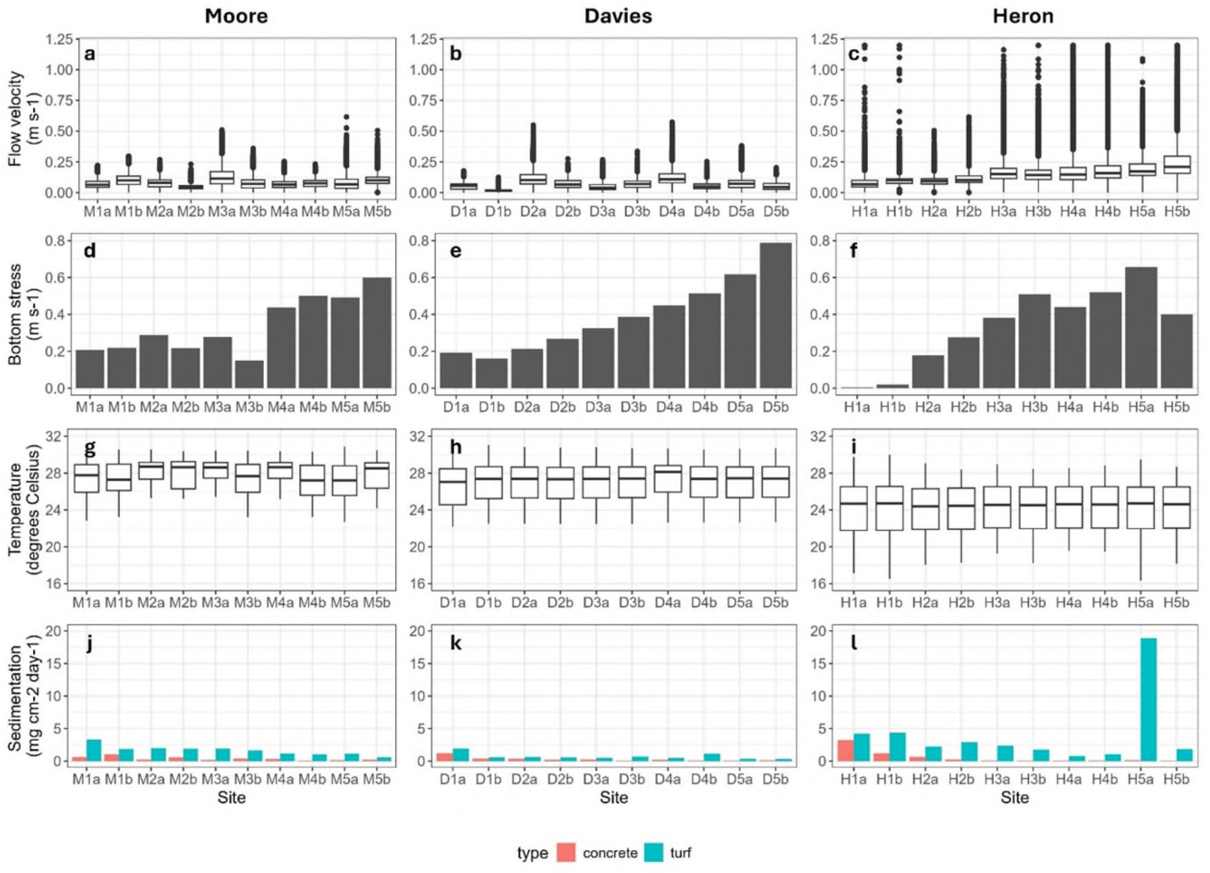

Figure S1. Summary of environmental variables across sites at Moore, Davies and Heron Reefs. Panels show: (a-c) boxplots illustrating the median and variability of *in-situ* flow velocity at each site; (d-f) bar graphs summarizing site averages of modelled long-term bottom stress predictions; (g-i) boxplots showing the median and variability of *in-situ* temperature; and (j-l) bar graphs summarizing site averages of sedimentation rates on turf and concrete surfaces.

Figure S2

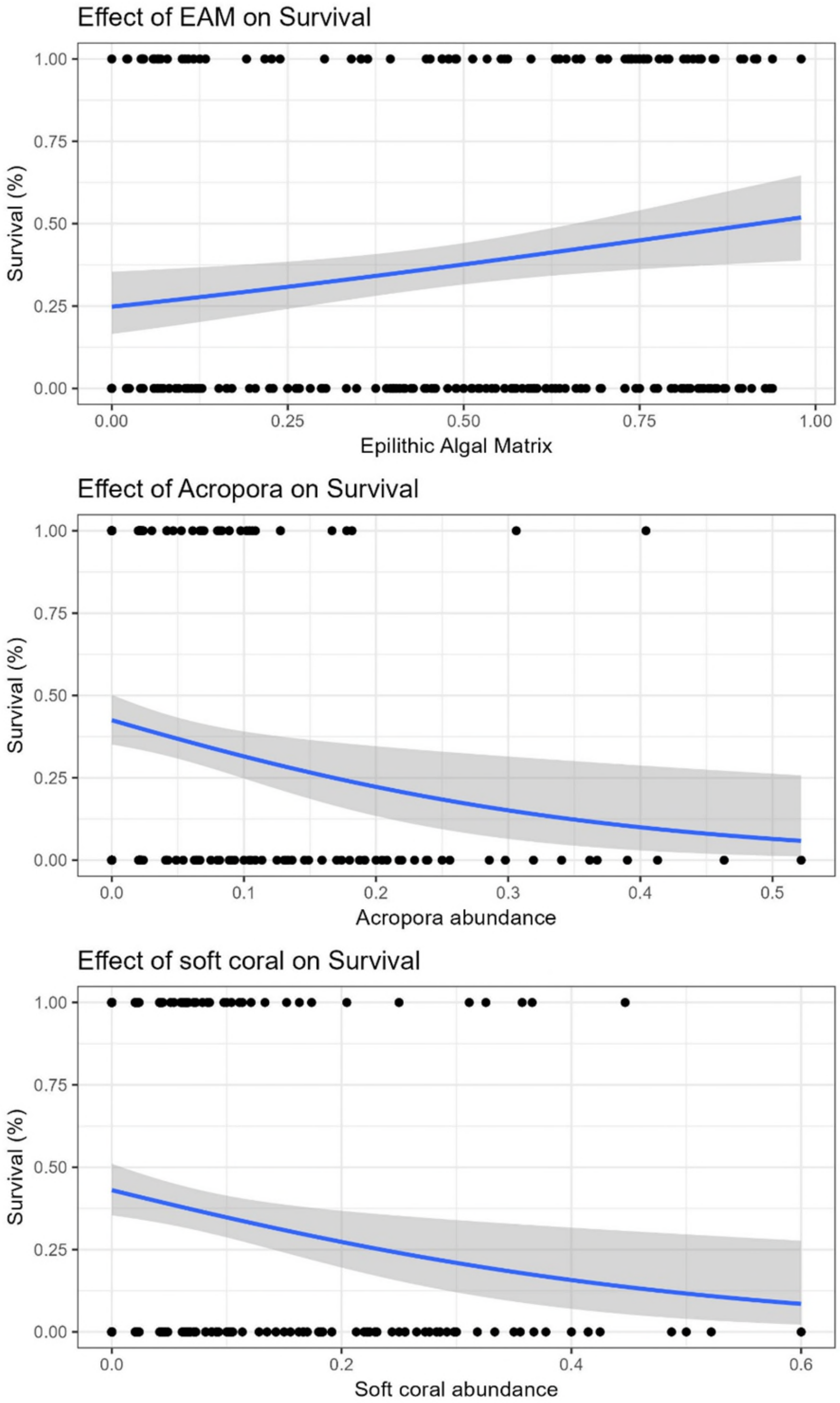

Figure S2. Relationship between EAM, Acropora and soft coral abundance and coral survival at **Moore Reef** following a logistic regression analysis. In blue is the predicted probability of survival as a function of key benthic group abundance, and the shaded area indicates the 95% confidence interval.

Figure S3

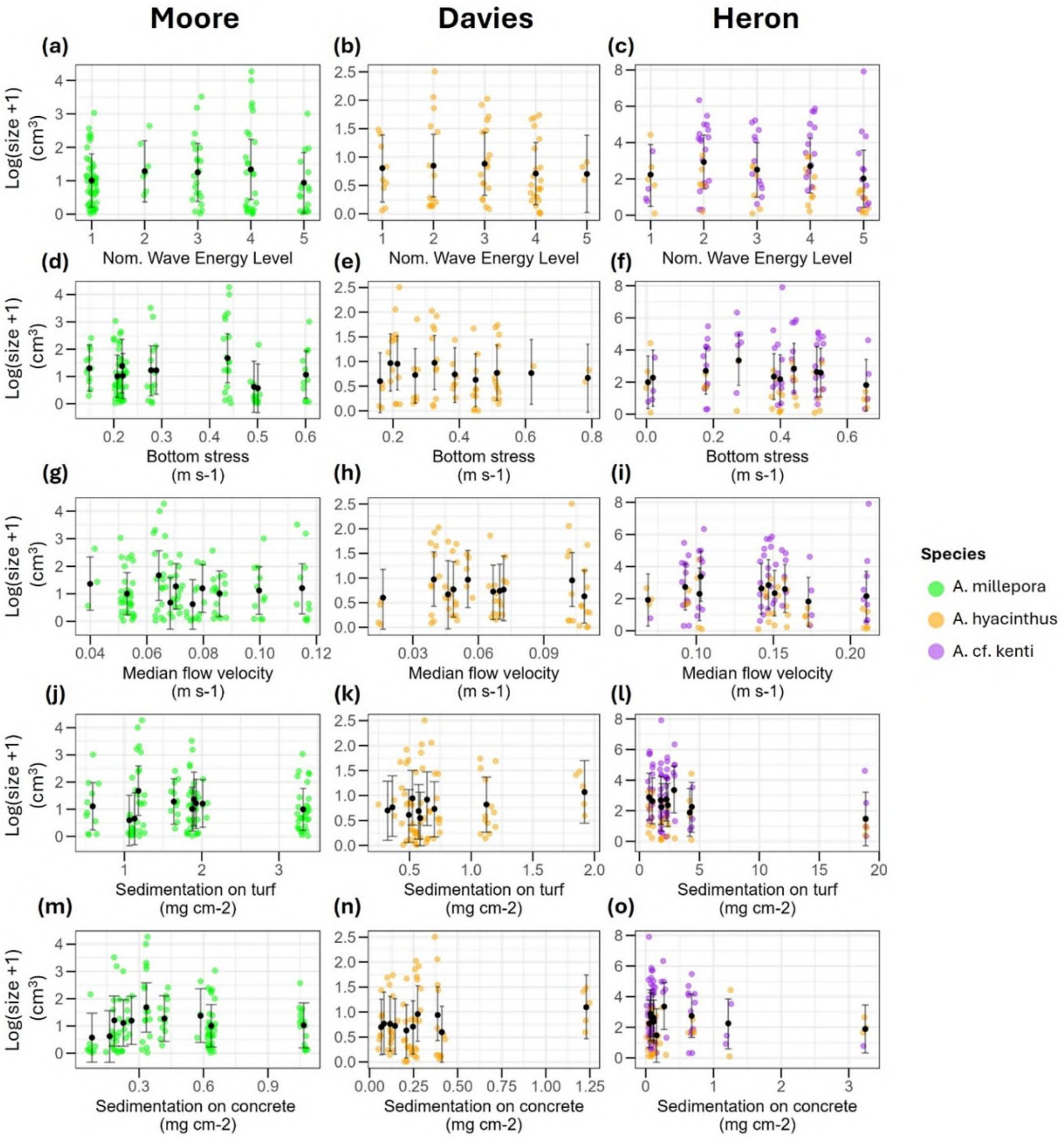

Figure S3. Mean predicted size (log+1 transformed, in cm^3) across Moore, Davies, and Heron Reefs in relation to: (a-c) nominal wave energy level predicted by spatial models; (d-f) long-term bottom stress predictions; (g-i) median flow velocity measured *in-situ* at each site; (j-l) sedimentation on turf pods; (m-o) sedimentation on concrete sed pods. The black points represent the mean predicted size with their 95% credible intervals.

Figure S4

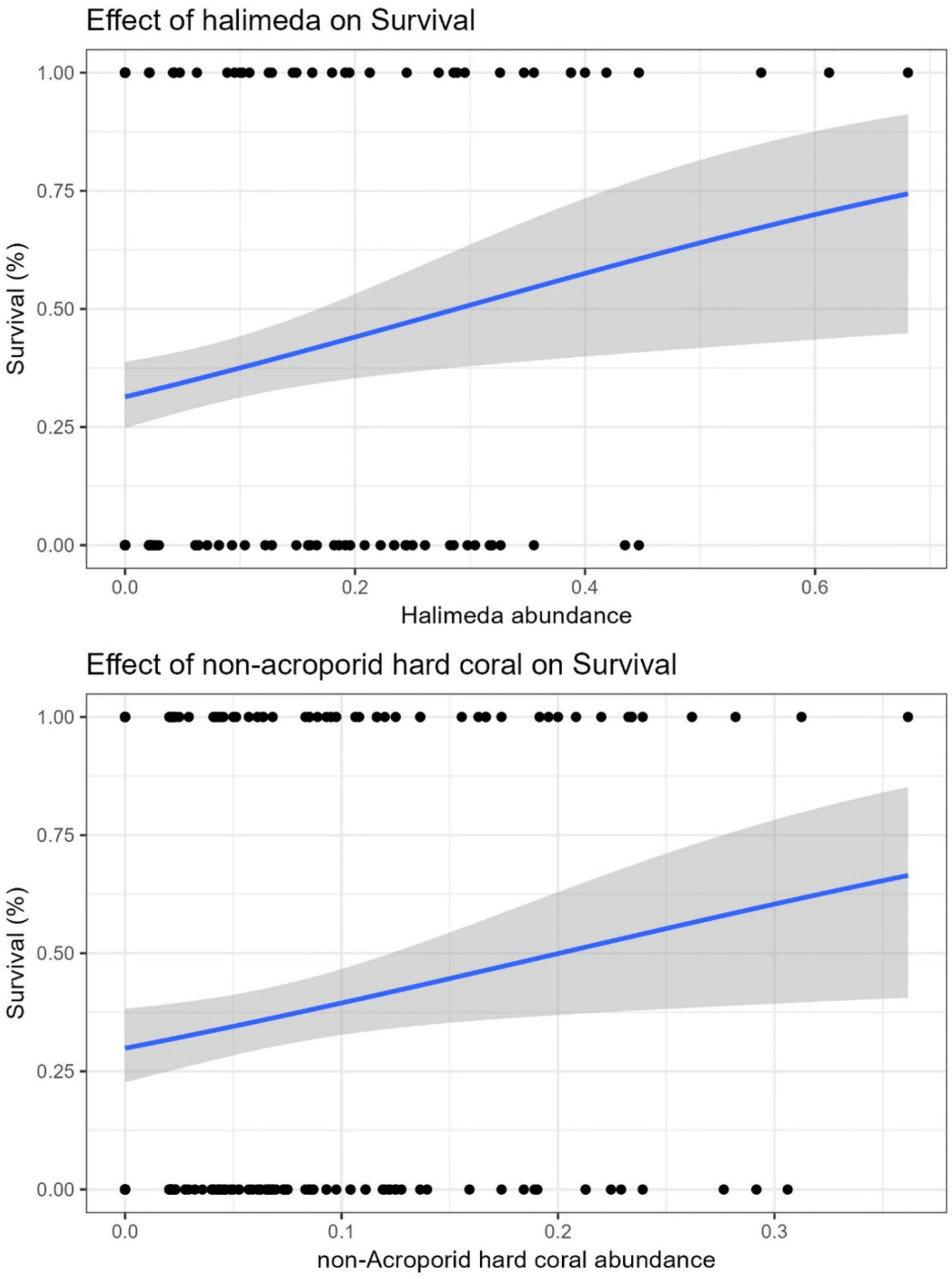

Figure S4. Relationship between halimeda and non-Acroporid hard coral abundance and coral survival at **Heron Reef** following a logistic regression analysis. In blue is the predicted probability of survival as a function of key benthic group abundance, and the shaded area indicates the 95% confidence interval.

Figure S5

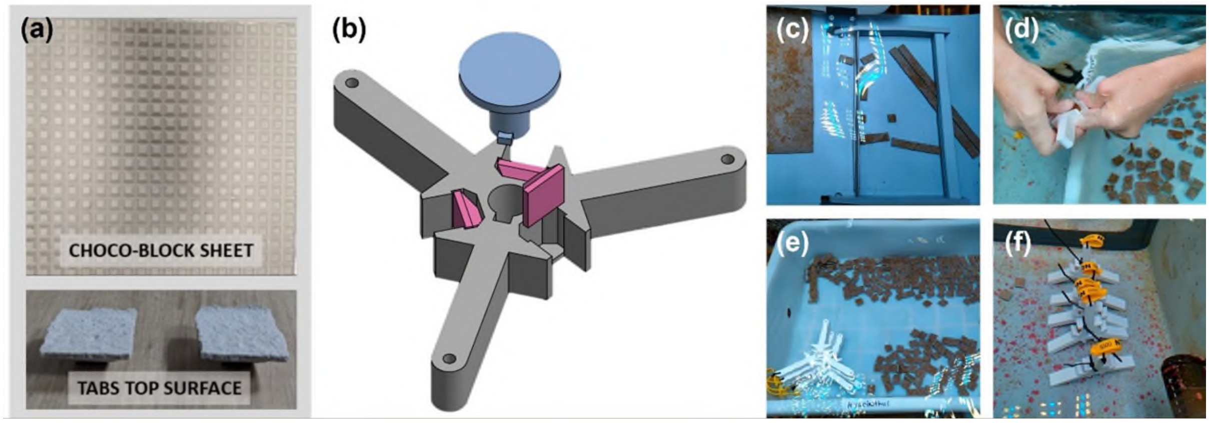

Figure S5 (a) Settlement sheet made of concrete and mortar, displaying the top surface and individual tabs. **(b)** Schematic representation of the seeding device made of moulded 95% Alumina ceramic (fabricated by Shanghai Gongtao Ceramics CO., Ltd. PRC) which exhibits high chemical inertness and long-term durability with no toxic effects to the marine environment [12]. **(c)** Settlement sheets were fragmented into tabs using a blunt slicer while submerged. **(d)** Tabs were slotted into the tab placeholders and secured with a 3D printed plastic cap glued on top of the device. The cap was further secured using a zip-tie looped around the device and through the centre hole of the device and cap. **(e)** Species and tabs were selected for insertion into the devices, keeping species strictly separated. **(f)** Assembled seeding devices with an ID tag attached to one arm of each device, which also served as a reference point for identifying individual tabs.

References

1. Radford, B. *et al.* A remote sensing model for coral recruitment habitat. *Remote Sensing of Environment* **311,** 114231 (2024).
2. Callaghan, D. P., Leon, J. X. & Saunders, M. I. Wave modelling as a proxy for seagrass ecological modelling: Comparing fetch and process-based predictions for a bay and reef lagoon. *Estuarine, Coastal and Shelf Science* **153,** 108–120 (2015).
3. Stevens, D. L. & Olsen, A. R. Spatially balanced sampling of natural resources. *Journal of the american Statistical association* **99,** 262–278 (2004).
4. Field, M. E., Chezar, H. & Storlazzi, C. D. SedPods: a low-cost coral proxy for measuring net sedimentation. *Coral Reefs* **32,** 155–159 (2013).
5. Tebbett, S. B. *et al.* Bio-physical determinants of sediment accumulation on an offshore coral reef: A snapshot study. *Science of The Total Environment* **895,** 165188 (2023).
6. Bürkner, P.-C. Bayesian Item Response Modeling in R with brms and Stan. *J. Stat. Soft.* **100,** 1–54 (2021).
7. Stan Development Team. RStan: the R interface to Stan. (2023). at <https://mc-stan.org/>
8. Hartig, F. DHARMa: Residual Diagnostics for Hierarchical (Multi-Level / Mixed) Regression Models. (2024). at <https://CRAN.R-project.org/package=DHARMa>
9. Wickham, H. *ggplot2: Elegant Graphics for Data Analysis.* (Springer-Verlag New York, 2016). at <https://ggplot2.tidyverse.org>
10. Kassambara, A. & Mundt, F. factoextra: Extract and visualize the results of multivariate data analyses, R package v. 1.0. 7. *factoextra: Extract and visualize the results of multivariate data analyses, R package v. 1.0. 7* (2020).
11. Oksanen, J. F. *et al.* vegan: Community Ecology Package. (2022). at <https://CRAN.R-project.org/package=vegan>
12. Fong, J. *et al.* Effects of material type and surface roughness of settlement tiles on macroalgal colonisation and early coral recruitment success. *Coral Reefs* **43,** 1083–1096 (2024).
